# The Teeth of Our School Children; What Can Be Done to Save Them?

**Published:** 1895-02

**Authors:** J. C. McCoy

**Affiliations:** Santa Ana, Cal.


### The Teeth of Our School Children; What Can Be Done To Save Them?

BY J. C. MCCOY, M.D., SANTA ANA, CAL.

Read in the Section on Dental and Oral Surgery, at the Forty-Fifth Annual Meeting of the American Medical Association held at San Francisco, June 5-8, 1894.

No doubt this question has been asked by every intelligent practitioner, as day by day he looks into the oral cavities of Young America, seeing there the almost universal dissolution of the masticatory organs. Those who live in small cities or towns and practice for all classes of society, have a better opportunity to know the condition of the teeth of the masses than does our city brother with his aristocratic patients.

A large majority of the people can not pay for skilful operations, and their teeth must be saved in an inexpensive manner or be gradually lost before middle age, to be replaced by miserably fitting rubber dentures, with the accompanying shrunken alveola and uvula-like appendages called gums, condemned to worry through the remainder of their earthly existence feeling that nature did a poor job on their masticatory organs, or they would have lasted till old age, irrespective of ignorance and neglect from their earliest childhood. Ignorance and neglect go hand in hand, and are the cause of more diseases and of the loss of more teeth than all other causes combined.

For the past ten years my practice has been in a community that numbers among its citizens many persons of culture and refinement. Others whose exterior apportionments of life are all that could be desired, and whose children are being trained in all the arts and sciences of the day except that of cleanliness of the mouth. We have thousands of this class. I have examined and worked for hundreds of them in the past ten years. The ignorance on the part of the parents, and neglect on the part of the children who know better, is one of the wonders of the nineteenth century.

In one school of 700 pupils, 500 from 10 to 18 years of age, I distributed printed slips with the following questions: Do you cleanse your teeth with a brush every day? Do you cleanse your teeth with a brush twice a day? The teachers requested the pupils to answer the questions by writing the word, yes or no, to each question. The slips were immediately gathered up. On summing up, it was ascertained that out of 500 pupils, 50 cleaned their teeth twice a day; 275 used the brush sometimes; while 175 did not own a brush. Notice, the ages were from 10 to 18. In the primary department of 200 pupils, from 6 to 10 years of age, the teachers said they did not think there were 10 children in the department who used a toothbrush.

This school is not an exceptional one in this matter, as further inquiry and investigation demonstrated. In fact its graduates take high rank at our universities, and if there is any difference, it is in advance of most schools in percentage of those who have clean mouths, as well as neat clothes and bright faces.

When there is so much neglect, and so little real care of the mouth, it is not at all strange that the sixth year molars have to be sacrificed daily, because the parents can not go to the expense of treatment to have them preserved, thinking all the time that this most valuable tooth is deciduous, and soon to be replaced by one that is bacteria proof and will last forever, in a mouth that has never been properly cleaned.

The school of 700 pupils mentioned, where only 50 made any pretence to regularly care for the teeth, shows what a field for instruction and training every teacher has. What an opportunity for philanthropy and missionary work!

Our children's teeth must be saved. Experience has taught us that it is impossible to repair the ravages of decay, except in a limited degree. Prevention through cleanliness and proper care of the teeth is the only way possible and practicable to limit the wholesale destruction. Yes, I say limit, for even when ordinary care is used there is still room for the work of tbe skilful dentist.

The question before us is: What can we do to save the teeth of our school children?

American dentistry leads the world to-day, and the world justly honors us for the great advances we have made in the preservation of teeth but the fact still confronts us that millions of teeth are annually lost in America, that need not have been sacrificed if proper care and cleanliness of the mouth had been begun in childhood and continued to manhood and womanhood.

The dentists of America, a noble and philanthropic band of 20,000, have done much to educate and train our citizens in the care of their teeth. We must do more. And at the same time in order to multiply our usefulness, we must solicit the aid of our public school teachers. With their co-operation we can reach and train millions of children.

A majority of the children of our land are in our public schools. They are under the teachers' instructions from 5 to 17 years of age. If the teachers were required to instruct and train the children in the proper care of their teeth, and to insist upon their carrying out such instructions practically at home, we would have accomplished—or at least begun—a great work. If such pupils could be trained from infancy up through all the grades to the high school, I am very sure we would see men and women with better teeth than the average American of to-day. The teachers are the only ones who can do this work. The parents of a large per cent. of the pupils are ignorant and careless almost beyond belief, and their children will follow their footsteps, unless we dentists of America come to the rescue.

How can we bring this matter prominently before the educators of our land and secure their hearty co-operation?

I hope this Association will pass resolutions on the subject, indorsing the plan, and by its sanction give prominence to its importance.

Let the dental and medical press advocate it. Let each State Dental Association not only pass resolutions on the subject, but appoint a competent committee to arrange a manual on the subject of the care of the teeth. Let the same committee induce the State Board of Education to adopt such manual as a textbook to be used by teachers, and taught in our normal schools, requiring teachers to be able to pass an examination upon the contents of such manual, and then require them to carry out such instructions in their respective schools. May each one of us constitute himself into a special committee, to see that the spirit of this paper is carried out practically in the schools of our neighborhood.

It is not necessary for me to give more than a few hints on the subject to this body of intelligent dentists before whom I have the honor to speak. I hope to gain not only your hearty approval to this plan, but your enthusiastic co-operation, so that inside of twelve months every public school in our broad land will have begun the much needed reform of cleanliness of the teeth and mouth. So that in the future examinations of schools on the subject of oral hygiene, instead of there being only 50 out of 700 who clean their teeth regularly, we will not find 50 careless ones in 1,000.

This is a great work. We alone can do it. Let each one of us resolve to do our full share and prove ourselves philanthropists to the rising generation.

